# Targeting K-Ras and apoptosis-driven cellular transformation in cancer

**DOI:** 10.1038/s41420-021-00457-5

**Published:** 2021-04-14

**Authors:** Isha Godwin, Nikhil Ponnoor Anto, Smitha V. Bava, Mani Shankar Babu, Goodwin G. Jinesh

**Affiliations:** 1 Saveetha Medical College, Thandalam, Chennai, Tamil Nadu 602105 India; 2 Shraga Segal Department of Microbiology, Immunology and Genetics, Ben-Gurion University of the Negev, Beersheba, Israel; 3 Department of Biotechnology, University of Calicut, Malappuram, Kerala 673635 India; 4 Department of Botany, University College, Thiruvananthapuram, Kerala 695 034 India; 5 Departments of Molecular Oncology, and Sarcoma, H. Lee Moffitt Cancer Center & Research Institute, Tampa, FL 33612 USA

**Keywords:** Apoptosis, Cancer stem cells, Target identification, Cancer stem cells, Cancer

## Abstract

Cellular transformation is a major event that helps cells to evade apoptosis, genomic instability checkpoints, and immune surveillance to initiate tumorigenesis and to promote progression by cancer stem cell expansion. However, the key molecular players that govern cellular transformation and ways to target cellular transformation for therapy are poorly understood to date. Here we draw key evidences from the literature on K-Ras-driven cellular transformation in the context of apoptosis to shed light on the key players that are required for cellular transformation and explain how aiming p53 could be useful to target cellular transformation. The defects in key apoptosis regulators such as p53, Bax, and Bak lead to apoptosis evasion, cellular transformation, and genomic instability to further lead to stemness, tumorigenesis, and metastasis via c-Myc-dependent transcription. Therefore enabling key apoptotic checkpoints in combination with K-Ras inhibitors will be a promising therapeutic target in cancer therapy.

## Introduction

Cellular transformation is an important process in tumorigenesis^1–3^. The ability to induce cellular transformation was initially considered as the characteristic feature of oncogenic viruses and then was narrowed down to the individual but multiple oncogenes^4–9^. Cellular transformation which drives evasion of apoptosis, phagocytosis, and accumulation of genomic instability can result in tumorigenesis^3,10,11^. A huge body of evidence indicates that transcription-driven epithelial to mesenchymal transition in combination with cell death evasion is the main cause for metastasis^12^, a pivotal event that leads to mortality. Thus, there is a pressing need to understand the pivotal players of cellular transformation and the ways to combat cellular transformation for therapeutic purpose.

**Fig. 1:**
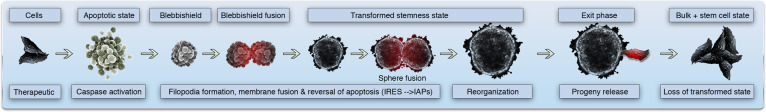
Overview of blebbishield emergency program. Schematic showing the major steps involved in K-Ras-driven cellular transformation by blebbishield emergency program.

Cellular transformation is a process in which the cancer cells (or normal stem cells) acquire the habit of aggregation, cell fusion, and growth into spheroids^13–16^. Mere aggregation of cells is not sufficient to complete cellular transformation and this process needs cell fusion through membrane lipids such as cholesterol^14^. Cellular transformation^15^ is also mentioned in literature with different names such as spheroid formation^17^, clonogenic growth^18^, focus formation^19,20^, bullet formation^21^, and so on. Recent studies envisaged that the blebbishield-mediated transformation^3,11,12,14,22–26^ drives cellular transformation after induction of apoptosis (Fig. 1). If the cells undergo transformation for the first time from normal cells which is accompanied by alterations from normal cell behavior, it is referred to as neoplastic transformation^27^ and it leads to tumorigenesis depending on the extent of genomic instability it has accumulated^11^. However, if a cancer stem cell (that is already transformed but not in a transformed/spheroid state) undergoes more rounds of cellular transformation, then it is referred to as malignant transformation^3,11,14,22,26^ because these additional rounds of transformation often results in further increase in genomic instability and metastasis^3^. It has been repeatedly shown that cells capable of transformation are able to form tumors in xenograft models and that the cellular transformation activity distinguishes cancer stem cells from bulk cancer cells^3,11,14,22,26^. Recent studies have materialized the fact that blebbishield emergency program, which includes apoptosis induction and spheroid formation were inevitable steps of K-Ras-driven cellular transformation^3,11,14,22,24,26^. However, the studies on blebbishield-mediated transformation envisaged that spheroid growth is not a permanent feature of cellular transformation and the spheroids can eventually give rise to polarized monolayer of cancer cells using the exit phase of blebbishield emergency program^3,14,26^ (Fig. 1). At this point, the cells may have lost the transformed state to various degrees but may retain stemness depending on the stemness regulatory transcription factors present. In blebbishield emergency program, the colonies that exited from spheroid-transformed state express increased c-Myc, a stemness transcription factor^14^. Furthermore, under certain circumstances that unleash the apoptotic/cell death process, these cells regain the ability to form spheroids^28^ depending upon death ligands and death receptors involved^24^. In the case of blebbishield emergency program, c-Myc has been shown to undergo a transient downregulation during the apoptotic phase^14,24^. In this context, lethal death receptor signaling is suppressed by K-Ras signaling to favor cellular transformation and metastasis^29^.

Here we draw evidences from the literature on cellular transformation to shed light on the key players that are required for K-Ras-driven cellular transformation that is coupled to apoptosis. Based on the available literature we also discuss that the defects in key apoptosis regulators such as p53 and mitochondrial apoptosis are key to apoptosis evasion, cellular transformation, and genomic instability and discuss the therapeutic vulnerability points that can be exploited for future drug discovery. Of note, many agents discussed in this review are in fact used to understand the therapeutic vulnerable points of K-Ras driven cellular transformation through blebbishield emergency program and therefore are not to be used for clinical purposes without clinical trials.

## Molecular targets of cellular transformation in cancer

**Fig. 2:**
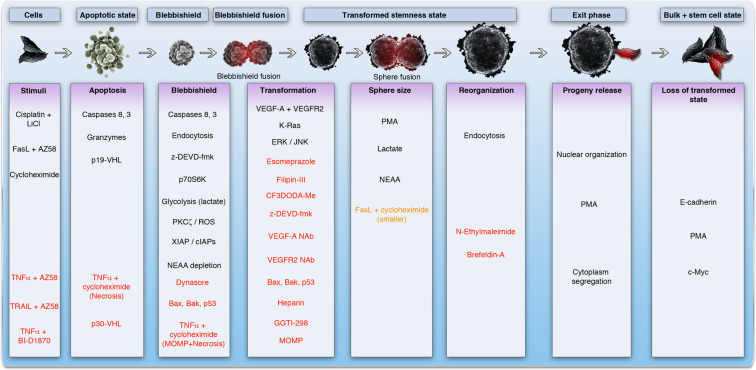
Regulators of blebbishield emergency program. Schematic showing the positive (black font) and negative regulators (red font) of major steps involved in K-Ras-driven cellular transformation by blebbishield emergency program. LiCl lithium chloride, AZ-58 Smac mimetic, NEAA non-essential amino acids, MOMP mitochondrial outer membrane permeabilization, PMA phorbol 12-myristate 13-acetate.

K-Ras driven cellular transformation through blebbishield emergency program has a well-documented apoptotic phase, transformation phase, and an exit phase^3,14,22–26,30–34^ (Fig. 1). Multiple agents that promote or inhibit these phases were identified to date (Fig. 2). Of which, agents that promote or inhibit the transformation phase are very important as this step of blebbishield emergency program can undermine the therapeutic elimination of cancer cells through apoptosis. Vascular endothelial growth factor (VEGF) signaling is a major driver of transformation phase^14,22,26^ despite epidermal growth factor (EGF) is being widely implicated^35,36^. The ability of EGF to induce VEGF secretion^37^ indicates that EGF might work through VEGF signaling to induce cellular transformation in addition to its role in DNA repair^11^. Likewise, many receptor tyrosine kinases influence cellular transformation suggesting there should be a central node where these signaling must converge. K-Ras acts as that central node in many instances under the stimulation of multiple RTKs^11^ (Fig. 2). VEGF mediates cellular transformation under the influence of mutation-activated K-Ras^38^ and targeting K-Ras activation inhibits VEGFR2 expression and abrogates transformation after apoptosis by blebbishield emergency program^24,34^. Thus, various RTKs and VEGF might act redundantly. K-Ras, VEGF-A, VEGFR2, p70S6K, FoxM1, Raf-1, ERK-1/2, and p90S6K are directly or indirectly linked to VEGF-mediated cellular transformation. An alternative to the notion that multiple RTKs might converge on K-Ras, different RTKs may play a dominant role depending on the cancer types of tissue/cell type (luminal, basal, epithelial, mesenchymal, etc.^39^) or even use other Ras family members such as N-Ras^40^ and H-Ras^41^. Ras especially K-Ras in collaboration with p47^phox^ and PKC-ζ (and possibly other isoforms of PKC) generates reactive oxygen species (ROS) resulting in sustained activation of PKCs^11,25,42^ (Fig. 2). PKCs in turn activate p70S6K to promote IRES translation of critical targets that regulate stemness (c-Myc/N-Myc), survival (XIAP, cIAP-1,-2,) to prevent apoptosis^11,23,24,26^ (Figs. 1 and 2). Notably, K-Ras^11^ and JNK are implicated in both cellular transformation^43^ and cell death^44^ reiterating the point that, cellular transformation is tightly linked to life and death decisions of the cancer cell (Figs. 1 and 2).

## Role of blebbishield emergency program in cellular transformation

The next important phase that could be therapeutically exploited is the apoptotic phase. For centuries, apoptosis was considered as the last chapter of cells. It was not known until recently that cancer stem cells can survive after the commitment of morphological and biochemical apoptosis. Apoptosis is an essential intermediate step in K-Ras-driven cellular transformation^11,24^. Therefore, the apoptotic execution is overwhelmed by survival signals at multiple points to initiate the resurrection process to facilitate cellular transformation (Fig. 2). The main events that are overwhelmed during blebbishield-mediated transformation of cancer stem cells are (1) protection of mitochondria from outer membrane permeabilization (MOMP)^23,24^ which is primarily done by ROS detoxification systems, (2) protecting or translating the IRES anti-apoptotic target molecules^23,24,45,46^ including XIAP, c-IAP1, c-IAP2, and so on, (3) overriding secondary necrosis, [a process that follows apoptosis as a result of glycolytic and tricarboxylic acid (TCA) cycle shutdown leading to the paucity of ATP]^24,47,48^, (4) establishing VEGF autocrine loop through ROS and internal ribosome entry site (IRES) translational elements^11,24,26^, (5) overriding chromosomal instability checkpoints^3^ (mainly by suppressing p53-dependent checkpoints), and (6) overriding immunological and phagocytosis checkpoints^3^ (by promoting galectin-3). K-Ras plays a central role in regulating all these six main events^3,11,24^, albeit it has been shown in different contexts in addition to blebbishield emergency program.

Cellular transformation is achieved by inactivating Bax and p53-dependent apoptosis^49^. Selective suppression of p53 happens during the transformation step of blebbishield emergency program^3^. Cleavage of Bax by proteases into p18-Bax damages the mitochondria by MOMP and cells with MOMP were unable to transform by blebbishield emergency program^23,24^ (Fig. 2). Notably, Bax-p18 is a more potent mitochondrial outer membrane potential inducer than full-length Bax^50^. Interestingly, Bax and Bak deficiencies are linked to cellular transformation, demonstrating the importance of Bax and Bak in preventing cellular transformation after apoptosis^49,51^. The tumor suppressor p53, a well-known inducer of apoptosis is known to suppress transformation^52,53^ as well as known to transform cells^54^ depending on Bax status^49^. Hence Bax-p18 plays an important role downstream of p53 in preventing transformation after the commencement of apoptosis. This is because Bax is a p53 target gene^55^.

**Fig. 3:**
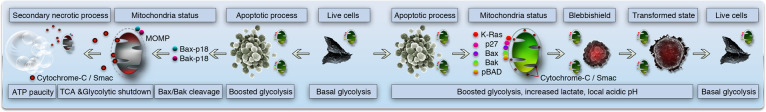
Secondary necrosis marks proper apoptosis and abrogation of transformation from blebbishields. Schematic showing the positive and negative regulation of secondary necrosis during blebbishield emergency program in transforming cancer stem cells. Note that oligomers of K-Ras, pBAD (S-112), p27, Bax, and Bak are present in live cells and are promoted during apoptotic phase, but p18 forms of bax and bak are present only in MOMP and secondary necrosis accompanied by Smac and cytochrome-C release.

Secondary necrosis a process that occurs in apoptotic cells, spills the intracellular contents outside of the apoptotic cell and culminates in the abrogation of cellular transformation (Fig. 3)^24^. Secondary necrosis is mostly observed in vitro but it also happens in vivo^56^. Under in vivo conditions the apoptotic cells are cleared by phagocytosis before reaching the secondary necrotic stage. However, when massive number of cells undergo apoptosis that outnumbers phagocytes, or when apoptosis happens in phagocyte restricted areas of tissues, secondary necrosis can be detected^56^. Secondary necrosis is a clear indication of the glycolytic shutdown, or to be precise, the necrotic state is triggered by the paucity of intracellular ATP in apoptotic cells^48^. In blebbishield emergency program, apoptotic cells generate ATP continuously through oligomerization of K-Ras, BAD, p27, Bax, and Bak at mitochondria to boost glycolysis, which overrides secondary necrosis (Fig. 3)^24^. Oligomerization of Bax is implicated in MOMP induction and cytochrome-C release^57^, however, identification of Bax oligomers in addition to Bak, BAD, p27, and K-Ras oligomers in non-apoptotic cells convincingly links the oligomers to glycolytic function than to MOMP^24^. On the other hand, generation of p18-Bax and or p18-Bak is associated with secondary necrosis and abrogation of transformation from apoptotic cells^24^.

In addition to overriding secondary necrosis, apoptotic cancer stem cells also use IRES translation to neutralize pro-apoptotic signals^24^. Expression of p70S6K is one of the key targets which helps IRES translation by phosphorylating ribosomal S6 proteins^24^. Apoptotic cells are known to continue IRES translation^24,58^. Many of the IRES translational targets are strong anti-apoptotic molecules such as c-IAP1/2 (protects cells from extrinsic apoptosis^24,45,46^), XIAP (protects cells from caspase-3 mediated damage^45,46,59,60^), c-Myc (multiple survival and apoptotic functions), N-Myc (replenish ribosomal components by transcription^61^, protect mitochondria^62^, co-operates with Survivin during malignant transformation^63^ and drives blebbishield-mediated transformation after the induction of apoptosis^24^), BCL2 (protects mitochondria^57^), BCL_XL_ (protects mitochondria^57,64^), and so on.

Caspase-3 plays a dual role in cellular transformation. It is required for generating blebbishields by inducing apoptosis, however, inhibiting caspase-3 inhibits transformation through loss of N-Myc expression, suggesting that caspase-3 is required for IRES translation of N-Myc^24^. Notably, the degree of caspase-3 activation is important because full activation results in complete cleavage of PARP^24^ which can impair DNA repair mechanisms that are essential to reduce DNA-double strand breaks in the genome below the threshold of apoptosis induction. In this context, FasL in combination with Smac mimetic compound AZ-58 that result in partial caspase-3 activation and incomplete PARP cleavage results in cellular transformation after the commencement of apoptosis compared to the combinations of AZ-58 with TNF-α or TRAIL that has full caspase-3 activation and complete PARP cleavage^24^. Furthermore, Smac and cytochrome-C release from mitochondria can also influence caspase-3 and caspase-9 activation to determine the survival of apoptotic cancer stem cells^24^. In addition to N-Myc, VEGF-A is also an IRES translational target crucial for cellular transformation^26^, VEGF autocrine loop is necessary to drive transformation from blebbishields^26^. Reactive oxygen species (ROS) is known to induce VEGF expression^65,66^ through induction of base excision repair-mediated VEGF transcription^67^. Then VEGF-A protein expression is regulated by K-Ras/p47^phox^/PKC-ζ/p70S6K/IRES translation axis^11^. Phorbol 12-myristate 13-acetate (PMA) activates PKC-α and PKC-ζ through ROS to enhance VEGF mRNA stability^68^ and stimulates VEGF-A secretion to promote the exit phase of blebbishield-mediated transformation^26^. ROS is mainly produced through the p47^phox^ component of NADPH oxidase, which is modulated by the interaction of PKC-ζ with p47^phox^, and K-Ras^25^. Inhibiting ROS or inhibiting the expression of PKC-ζ and/or p47^phox^ abrogates blebbishield emergency program^25^ to reiterate the fact that ROS generation is crucial to establish the VEGF autocrine loop. Although excess ROS can stimulate p53 and induce proper apoptosis, the ROS have to be neutralized to promote cell survival through K-Ras stimulated antioxidant system such as PKC-ζ/PKC-ε/Nrf-2/HO-1 axis^11,69–71^. Hence ROS could play a double role to shift the balance either toward survival or death depending on the status of K-Ras/PKCs/Nrf-2/HO-1 axis activation^11^ and depolarization of mitochondrial membrane potential^23,72^.

**Fig. 4:**
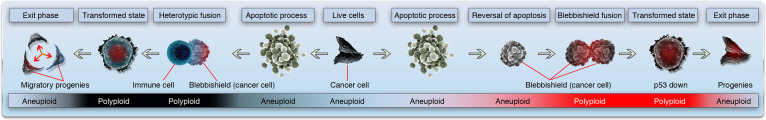
Evasion of apoptosis promotes ploidy level numeric genomic instability. Schematic showing the mechanisms of apoptotic and genomic checkpoint evasion by cancer stem cells through blebbishield emergency program. Note the self-fusion among blebbishields or blebbishield-immune cell fusion results in ploidy level chromosomal instability in cancer stem cells undergoing blebbishield emergency program. The dark shades in polyploid regions denote increased nuclear size and DNA content. The migratory progenies from blebbishield-immune cell hybrids are known to have high IGFBP5.

Accumulation of genomic instability (structural and numeric alterations in chromosomes) is a hallmark of transformed cells (Fig. 4). The degree of genomic instability is a potential indicator of the number of rounds the transformed cells evaded p53-directed genomic checkpoints. Overriding genomic checkpoints are primarily achieved by inactivating p53 by mutations or by suppressing p53 expression at critical stages of cell cycle or during apoptosis (Fig. 4). In the case of blebbishield emergency program, as the cells undergo more rounds of survival after apoptosis, p53 is suppressed and the chromosome number and nuclear size increases reflecting massive ploidy level numeric chromosomal instability (Fig. 4)^3^. This is primarily achieved by the fusion of apoptotic cells where the merged DNA from multiple apoptotic cells are pooled to a nucleoid state, which then reorganize into individual nuclei and subsequently into individual cells during the exit phase of blebbishield emergency program (Figs. 2 and 4)^3^.

When the apoptotic cells are capable of fusion, it fuses with immune cells rather than get phagocytosed by it (Fig. 4). This ability of apoptotic cancer stem cells is demonstrated both in vitro (by co-culturing immune cells with apoptotic cells) and in vivo (by introducing apoptotic cells into phagocytosis competent mice)^3^. Cancer stem cell immune cells hybrids were demonstrated both in vitro and in vivo (in hepatosplenomegaly). Notoriously, the hybrids acquired vigorous migratory behavior with high IGFBP5 expression (Fig. 4)^3^. Therefore, blebbishield emergency program orchestrates multiple aspects of tumorigenesis, immune evasion, and metastasis by directing cellular transformation.

## Targeting cellular transformation for cancer therapy

While blebbishield emergency program acts as the backbone of cellular transformation after apoptosis, many of the pivotal points of cellular transformation can be exploited as cancer therapeutic targets. Sp1 is a crucial transcription factor that regulates VEGF, VEGFR2 expression to regulate the K-Ras/ROS-driven VEGF autocrine feedback loop, and drives cellular transformation by blebbishield emergency program. In this context, impeding Sp1 node abrogates transformation^34^. It will be interesting to see if VEGF-trap^73^ designed to target angiogenesis could complement cytotoxic chemotherapeutics as combination therapy. Furthermore, FoxM1 inhibition targets cellular transformation by inhibiting VEGF expression^74,75^. At the protein level, heparin blocks VEGF-A to VEGFR2 binding thereby interferes with cellular transformation by abrogating blebbishield emergency program^14^ (Fig. 2). K-Ras inhibition also inhibits VEGFR activation^76^ and transformation^77^. In this context, K-Ras G12C mutant targeted inhibitors (AMG 510 and MRTX 849)^78,79^ or K-Ras G12D inhibitors (KS-58)^80^ could augment chemotherapy-induced apoptosis, in particular, it might inhibit survival after induction of apoptosis. K-Ras selectively suppresses p53 expression at protein level during transformation phase of blebbishield emergency program compared to apoptotic cells that are not able to undergo transformation^3^. This could probably happen through MDM2, an ubiquitin ligase that degrades p53^81^. Conversely, K-Ras inhibition enables p53 and downregulates MDM2^82^. In this context, agents such as K-Ras inhibitors or quercetin could be useful as these agents can target the K-Ras-directed suppression of p53^83^. Similarly, inhibition of the K-Ras-associated cascade ERK-1/2, JNK^84,85^, Raf-1, MEK-1/2^84^, AP1^85–87^ also impede or abrogate cellular transformation. In this context, MEK-1/2, ERK-1/2 inhibition with AZD6244 is demonstrated to augment cisplatin efficacy in K-Ras G12D mice background^88^. Ribosomal S6 kinases (p70S6K, p90S6K, and p52S6K) transduce survival signal downstream to K-Ras/PKC axis to drive IRES translation of vital survival molecules such as c-IAPs (c-IAP1/2, XIAP, c-Myc/N-Myc/Nrf-2, and so on). In this context, BI-D1870 (S6K inhibitor) has been shown to abrogate the transformation phase of blebbishield emergency program in combination with TNF-α. Furthermore, rapamycin, and CF_3_DODA-Me inhibit cellular transformation by inhibiting or degrading mTOR and p70S6K, respectively^34,89^. Apart from these agents, multiple drugs are known to target transformation phase of blebbishield emergency program (Fig. 2). However, many of these agents are not tested in combination with standard frontline therapeutics in the context of cancer therapy or not approved for human use. Agents like esomeprazole are already in clinic for other medical conditions and hence have fewer hurdles to be tested as combination agents.

Endocytosis plays a major role in blebbishield formation, transformation, and sorting membranes during the transformed sphere stage^26^. However, the precise targets that direct endocytosis during these processes have to be identified before aiming therapeutic targeting of endocytosis. Notably, K-Ras is a known driver of membrane reorganization and in turn, membrane reorganization activates K-Ras. N-ethylmalemide interferes with membrane reorganization (Fig. 2) but the use of N-ethylmalemide in the clinic is not feasible due to its high toxicity and non-selectivity. Therefore N-ethylmalemide is restricted to laboratory research alone.

The core apoptosis inducers such as p53, and Bax are potential targets to block cellular transformation. VHL enables p53 to promote apoptosis^90^, however, in the context of blebbishield emergency program, the p19-VHL and p30-VHL isoforms play oncogenic and tumor suppressor roles, respectively^33^. Enabling p53 holds the key to target mitochondria damage and inhibition of cellular transformation^52^. It is very important to know the mutation status of p53 because it affects the activation of caspase-3^91^, a pivotal trigger of blebbishield emergency program^24,26^. Defects in p53 could also deregulate miRNA-mediated regulation of tumorigenesis and metastasis because defective p53 is linked to chromosome 19 miRNA cluster (C19MC) in hepatocellular carcinoma^92^. Interestingly, p53 mutations cooperate with C19MC miRNA-520G to reverse interferon-γ signaling through CAAT enhancer-binding protein-β (CEBPB) in hepatocellular carcinoma^93^. Notably, miR-520G is accumulated ~3.75-fold more under transformed spheroid state than in monolayer growth conditions^93^. C19MC is also expressed in triple-negative breast cancer^94^, a known sub-type for therapy resistance.

## Conclusion

In conclusion, K-Ras-driven cellular transformation after apoptosis can be targeted by blocking vital signaling events (K-Ras, VEGF/VEGFR2, ERK-1/2, JNK, AP1, ROS, PKCs, p70S6K, IRES translation, Nrf-2/anti-apoptotic factor translation, and ROS neutralization) and by enabling mitochondrial apoptosis regulators such as p53 and Bax-p18. Importantly, K-Ras inhibition has the capability to enable *TP53* in cancers. Notably, the ability of cancer cells to generate p18-Bax is an essential aspect to abrogate transformation. Thus developing agents that target cellular transformation after apoptosis especially that are directed against K-Ras in combination with chemotherapeutics may help to combat aggressive therapy-resistant cancers in the future.
